# Mind Games: A Curious Case of Primary CNS Lymphoma Masquerading as Glioblastoma Multiforme

**DOI:** 10.7759/cureus.71358

**Published:** 2024-10-13

**Authors:** Tiana Junor, Nzube Ekpunobi, Jasjeevan Aujla

**Affiliations:** 1 Medicine, University of Medicine and Health Sciences, Basseterre, KNA; 2 Primary Care, SSM Health St. Mary's Hospital - Madison, Madison, USA; 3 Internal Medicine, Upstate University Hospital, Syracuse, USA

**Keywords:** central nervous system tumors, diffuse b-cell lymphoma, glioblastoma multiforme, neuro-oncology, primary cns lymphoma, surgical resection, t1-weighted imaging, tumor mimicry

## Abstract

The rare diffuse B-cell lymphoma subtype of primary central nervous system lymphomas (PCNSL) typically presents with nonspecific symptoms. Many of these symptoms overlap with those reported in cases of glioblastoma multiforme (GBM). However, despite the overlap in symptomatology, the two CNS tumors exhibit distinctly different imaging findings on MRI, which aids in diagnosis. Here, we present a rare case of the diffuse B-cell lymphoma subtype of PCNSL in the anterior right frontal lobe, initially diagnosed as GBM due to specific imaging findings. The diagnosis was corrected to PCNSL following biopsy and resection.

## Introduction

Diffuse large B-cell lymphomas (DLBCL), a subtype of non-Hodgkin lymphoma, account for 90% of the rarely occurring primary central nervous system lymphomas (PCNSL) [1]. DLBCLs predominate in PCNSL cases due to their aggressive nature and ability to infiltrate tissues beyond lymphoid organs, including the central nervous system [2]*. *Primary CNS lymphomas represent only 0.3-1.5% of CNS tumors [3]. Although rare, they can occur in both immunocompromised and immunocompetent individuals of either sex, with a higher prevalence in males [3]. Patients with primary CNS lymphoma usually present with nonspecific symptoms or focal neurological deficits corresponding to the location of the lesion [2]. Although uncommon, literature shows that certain glioblastoma multiforme (GBM) tumors can mimic the appearance of PCNSL on MRI, particularly due to similar enhancement patterns, which can lead to an initial misdiagnosis [4]. It is crucial to differentiate between PCNSL and GBM, as their treatments and prognoses differ significantly. The treatment for GBM typically involves complete resection, with an average survival rate of eight months. In contrast, a PCNSL lesion should be treated with induction therapy, offering a better prognosis of approximately 3.5 years for patients older than 50 [5]. We report a rare case of PCNSL in the anterior right frontal lobe, presenting with behavioral changes and initially misdiagnosed as GBM due to imaging findings that mimicked those of GBM.

## Case presentation

This case involves a 52-year-old Caucasian male with bipolar disorder who presented to the Emergency Department of a Southern Michigan teaching hospital, complaining of 10 days of intractable nausea and non-bloody, bilious vomiting. There was no association with food, and he denied abdominal pain or cramping. During the review of systems, he reported several months of a mild, intermittent throbbing frontal headache, which typically occurred in the morning and dissipated after a few hours. Occasionally, the headaches were accompanied by bilateral hand numbness and occasional bilateral shoulder or neck pain. There was no reported audio-visual aura. However, his wife noted recent behavioral changes, including worsening irritability, apathy, inappropriate responses to questions, and a flattening affect over several months.

**Figure 1:**
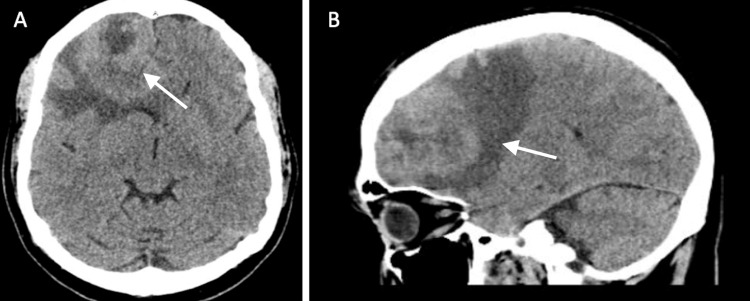
Contrast-enhanced computer tomography scan of the lesion A) Axial; B) Sagittal depicting a large partially necrotic mass in the anterior right frontal lobe

**Figure 2:**
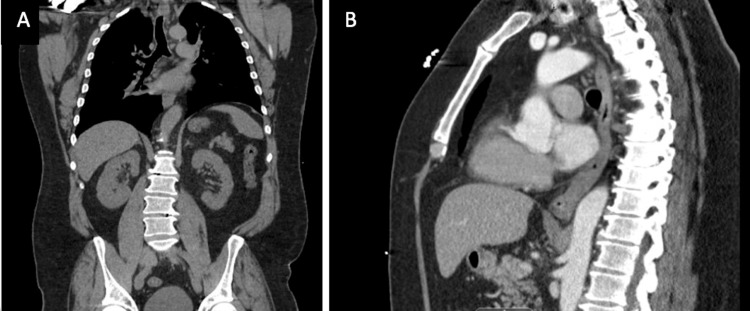
A) CT scan of the thorax abdomen and pelvis without contrast; B) CT scan of the thorax without contrast, depicting clear findings and no evidence of thoracic, abdominal, or osseous metastasis

**Figure 3:**
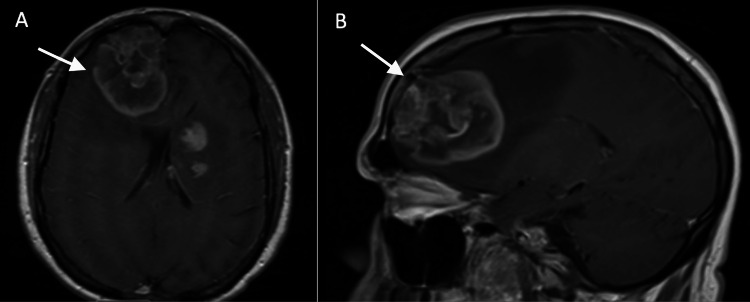
Contrast-enhanced magnetic resonance imaging of the lesion A) Axial T1 weighted image; B) Sagittal T1 weighted image depicting a large, heterogeneously enhancing 6 x 4.3 x 5 cm right frontal lobe mass with subfalcine herniation and 1 cm midline shift to the left

In the Emergency Department, a contrast-enhanced head CT scan revealed a large, partially necrotic mass in the anterior right frontal lobe, accompanied by surrounding vasogenic edema and localized mass effect (Figure 1). A CT scan without contrast of the thorax, abdomen, and pelvis was negative for metastatic disease (Figure 2). Contrast-enhanced MRI demonstrated a 6 x 4.3 x 5 cm heterogeneous mass with a 1 cm midline shift and subfalcine herniation, which was strongly suspicious for glioblastoma multiforme (Figure 3). The patient was started on IV steroids and levetiracetam for seizure prophylaxis and was admitted to the ICU for frequent neurological monitoring.

The Neurosurgery Unit evaluated the patient and transferred him to a tertiary center, where they successfully performed a right frontal craniotomy, microdissection, and gross total resection of the frontal mass. His postoperative course was uncomplicated, with no focal neurological deficits. He experienced resolution of both headaches and nausea before discharge. Pathology of the specimen revealed high-grade B-cell lymphoma, immunoreactive for LCA, CD-20, BCL-2, BCL-6, and MUM-1. The patient was discharged home in a hemodynamically stable and neurologically intact condition three days postoperatively, and he remained clinically stable with no residual deficits at subsequent follow-up.

## Discussion

Differentiating primary CNS lymphoma (PCNSL) from glioblastoma multiforme (GBM) is challenging due to overlapping clinical presentations. GBM often presents with signs and symptoms related to mass effect and cerebral parenchymal damage, including headaches, memory loss, nausea/vomiting, cognitive changes, personality changes, and seizures [6]. The clinical course for symptom presentation and progression is subacute, with neurological symptoms typically worsening insidiously over days to weeks [6]. Similarly, PCNSL presents with significant symptoms such as focal neurologic findings, headaches, nausea/vomiting, and ocular involvement [7]. Unlike patients with non-CNS lymphoma, who often exhibit classic B-symptoms like weight loss, night sweats, and fevers, these symptoms are uncommon in PCNSL patients, further complicating the diagnosis [2]. Due to the variable presentation of PCNSL, a high degree of clinical suspicion is necessary, particularly in immunocompetent patients. In the case of the 52-year-old patient, symptoms such as nausea, bilious vomiting, throbbing headaches, and behavioral changes did not provide sufficient clinical evidence to diagnose specific cancer, making imaging findings crucial for diagnosis.

Unusual imaging findings further complicate this case. Imaging is the primary non-invasive tool for diagnosing brain cancers, but a definitive diagnosis requires an invasive biopsy to guide treatment. Characteristic GBM MRI findings include a hypodense mass on T1 imaging with heterogeneous contrast enhancement, typically presenting as a tumor with a rim of enhancement and central clearing due to necrosis [8]. GBM is also associated with surrounding vasogenic edema, which appears as a hypodense T1 signal [8]. In contrast, PCNSL typically presents as a hyperdense or isodense tumor with surrounding hypodense vasogenic edema [8]. A key differentiator between PCNSL and GBM is that PCNSL usually shows homogeneous enhancement due to the general lack of internal calcifications, hemorrhage, or necrosis within the tumor [9]. Although rare, some studies, such as Buhring et al., have reported cases of PCNSL with heterogeneous tumors and varying degrees of necrosis [10]. Given the imaging findings in this patient, including partial necrosis within the tumor on CT and heterogeneous enhancement on MRI, diagnosing PCNSL was particularly difficult due to the rarity of such atypical imaging features.

Although the patient underwent a right frontal craniotomy with microdissection and gross resection of the frontal mass, a post-surgical biopsy revealed the tumor to be PCNSL rather than GBM. The complete resolution of symptoms, an uncomplicated postoperative course, and the absence of neurological deficits meant that the difference between the suspected and confirmed diagnosis did not significantly impact the patient's immediate outcome. However, the distinction between PCNSL and GBM is critical for determining appropriate treatment guidelines, especially in incomplete tumor resection or recurrence cases. GBM is primarily treated with surgical resection, whether complete or incomplete, followed by adjuvant radiation therapy to improve survival [7]. Systemic treatment, including alkylating agents like temozolomide, is also commonly used [7]. In contrast, PCNSL is treated with high-dose methotrexate as induction therapy, often combined with alkylating agents and rituximab to enhance tumor regression rates. The diagnosis of PCNSL not only affects the patient's future treatment but alters the current treatment approach. Even when presenting as a single mass on neuroimaging, PCNSL is often multifocal and infiltrative, so adjuvant therapy targeting any remaining cancer foci may be warranted in this patient [8].

## Conclusions

In conclusion, PCNSL tumors may initially resemble GBM tumors in imaging studies such as CT or MRI. Diagnosed GBM tumors that do not respond to initial treatment regimens may, in fact, be PCNSL tumors with imaging characteristics similar to those of GBM. Whenever feasible, a biopsy should always be performed to confirm the diagnosis and optimize treatment. Further research should focus on developing imaging techniques to more effectively differentiate atypical PCNSL tumors from GBM tumors.
